# Papulovesicular Riddle in an Atopic Individual

**DOI:** 10.7759/cureus.58102

**Published:** 2024-04-12

**Authors:** Mansi Satasia, Francis W Iacobellis, Lokesh Lahoti

**Affiliations:** 1 Internal Medicine, Saint Peter’s University Hospital, New Brunswick, USA; 2 Dermatology, New York-Presbyterian Hospital, New York, USA

**Keywords:** valcyclovir, herpes simplex virus type 1, eczema herpeticum, atopic eczema, head and neck atopic dermatitis

## Abstract

Atopic dermatitis (AD) characterized by pruritus and eczematous lesions makes individuals susceptible to various viral and bacterial infections. Eczema herpeticum (EH), also known as Kaposi's varicelliform eruption, is a severe herpes simplex virus infection that can be observed in individuals with AD. EH manifests with monomorphic vesicles and "punched-out" erosions accompanied by hemorrhagic crusts, primarily affecting eczematous areas. Misdiagnosis, often as impetigo, can lead to severe complications and even death. Timely diagnosis and treatment with acyclovir are crucial to avert these outcomes. Here we present a case of a 19-year-old male with AD who presented with a monomorphic vesicular rash.

## Introduction

Atopic dermatitis (AD) is a common chronic inflammatory skin condition characterized by pruritic lesions. A subset of AD patients experience eczema herpeticum (EH), a severe disseminated herpes simplex virus (HSV) infection with potentially life-threatening complications. EH typically presents as an eruption of monomorphic-shaped vesicles and may be accompanied by fever, malaise, and lymphadenopathy. It predominantly affects individuals with underlying skin conditions such as AD or other disorders leading to epidermal barrier dysfunction, including contact dermatitis, rosacea, pemphigus, and burns [1,2]. Left untreated, EH can result in severe complications such as secondary bacterial infections, keratoconjunctivitis, encephalitis, and meningitis [3]. Hence, early clinical identification and laboratory confirmation are crucial for prompt and targeted intervention.

## Case presentation

Clinical presentation

A 19-year-old male, an exam-going college student, presented at the emergency department with skin lesions accompanied by mild pruritus and a burning sensation for the past five days. It started as small red bump-like lesions around the left eye, which gradually spread around the right eye, forehead, and cheek over the subsequent 3-4 days. He was stressed due to upcoming exams and tried Benadryl and topical steroids but no relief. Interestingly, the condition worsened after a single application of topical steroids. He also reported dull headache across the frontotemporal area and excessive tearing from the eye, although no photophobia. His medical history includes asthma, AD, and allergies to shellfish, walnuts, jackfruits, pecan, and bacitracin. The other review of systems was unremarkable.

Cutaneous examination

**Figure 1:**
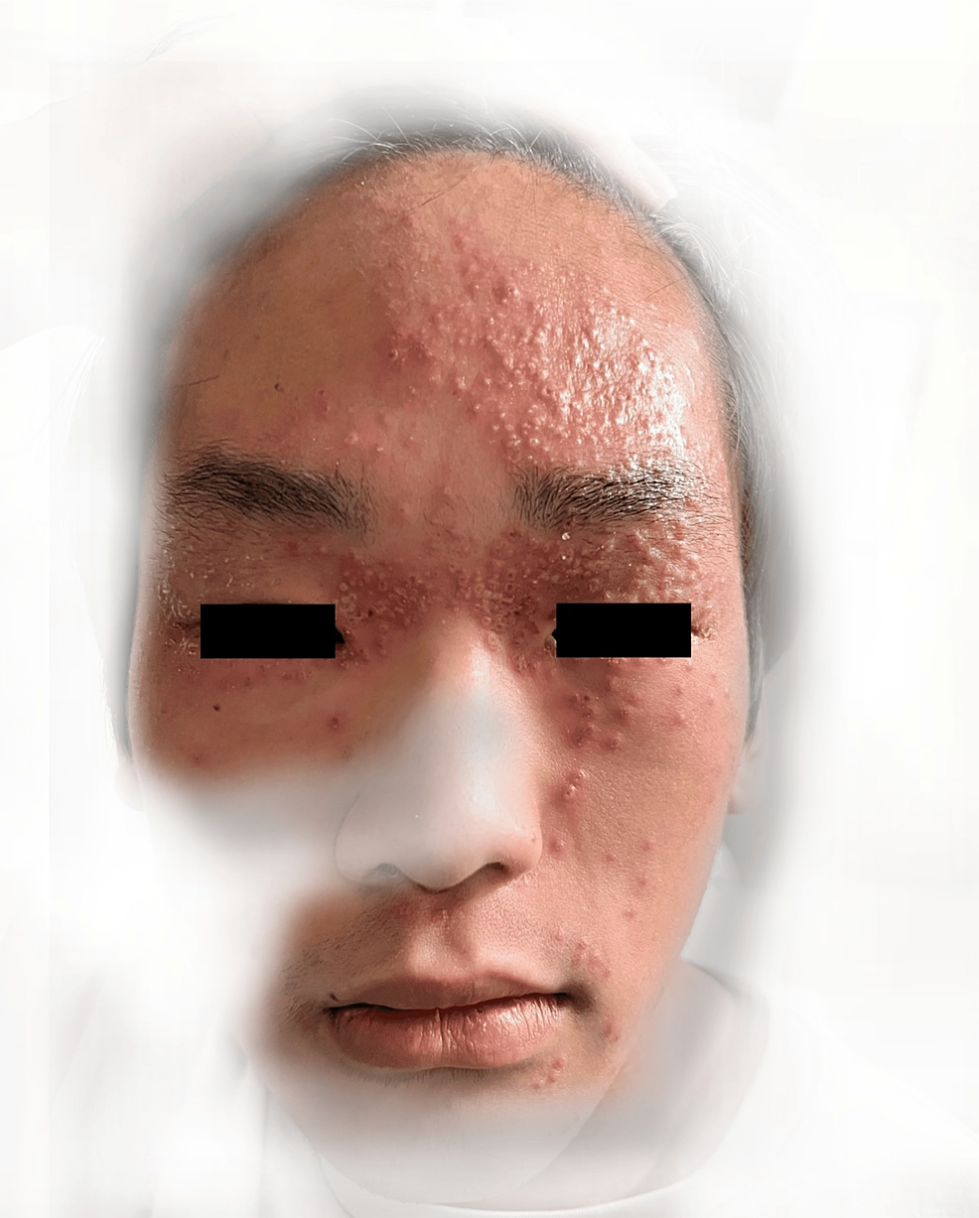
Multiple erythematous papulovesicular rashes predominantly surrounding both eyes, forehead, and left cheek, with some papules displaying central umbilication and crusting

There were multiple erythematous, monomorphic papulovesicular rashes predominantly surrounding the eyes, forehead, and left cheek, with some papules displaying central umbilication and crusting (Figure 1).

Management

**Figure 2:**
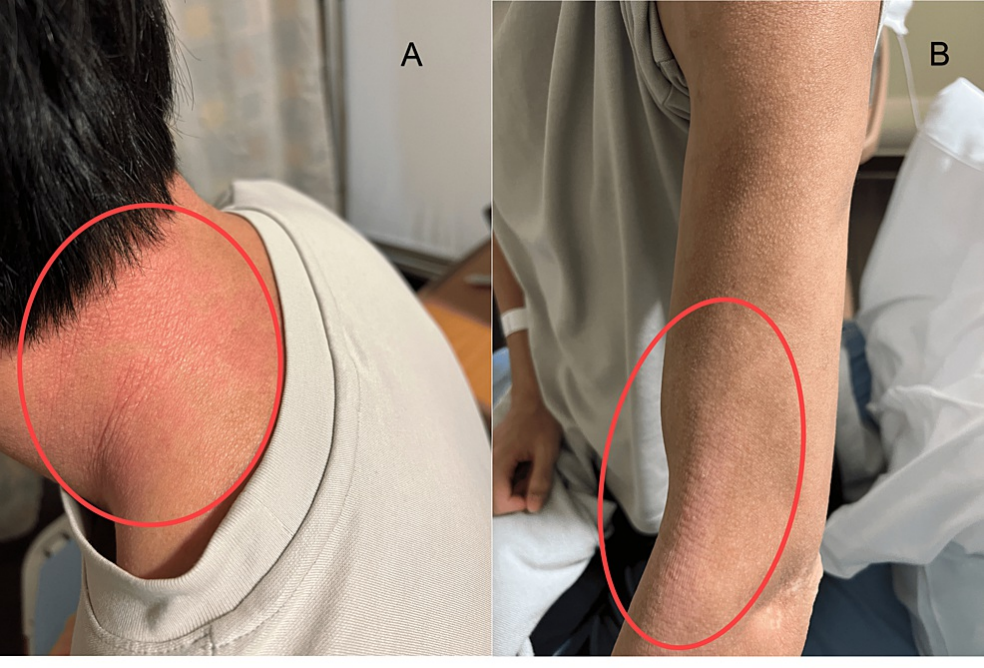
Atopic eczema on the nape of the neck (A) and lateral side of anti-cubital fossa of the left arm (B)

Routine blood work showed elevated eosinophils. Vesicular fluid was collected for antibody and viral marker analysis. Given his history of atopic dermatitis (Figures 2A, 2B) and the presence of a stressor, a provisional diagnosis of EH was made clinically.

**Figure 3:**
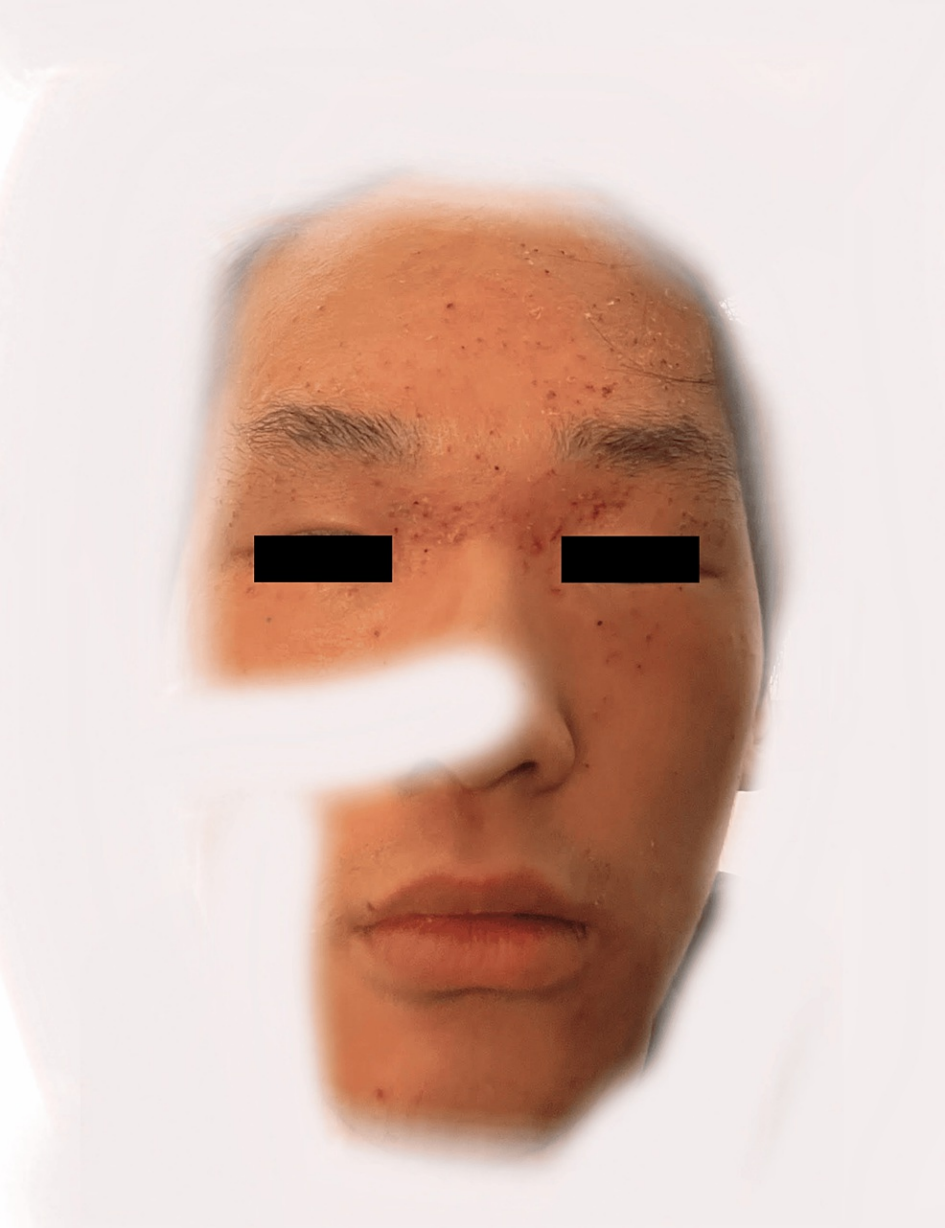
Improvement of skin lesions with valacyclovir treatment

Treatment with valacyclovir led to notable improvements within 24 hours and complete resolution of the skin lesions after continued treatment (Figure 3).

A single dose of 1 gm cefazolin was given prophylactically to prevent secondary bacterial infection. Subsequent laboratory analysis confirmed the clinical diagnosis. Vesicle fluid analysis revealed the presence of HSV type 1 by viral DNA PCR. Serological testing demonstrated positive HSV type 1 IgG antibodies, whereas HSV type 2 and varicella antibodies were negative.

## Discussion

AD, characterized by pruritus and eczematous lesions, makes individuals susceptible to various viral, fungal, and bacterial infections [1]. HSV, molluscum contagiosum virus (MCV), human papillomavirus (HPV), coxsackieviruses, and vaccinia virus (VV) can cause disseminated rashes in AD patients.

EH, first described by Moritz Kaposi in 1887, affects less than 3% of AD patients and exhibits sudden eruption of monomorphic vesicles and punched-out erosions with hemorrhagic crust over the eczematous area, often accompanied by fever and malaise [2]. The most commonly affected areas include the head, neck, and thorax. Although EH typically originates in skin affected by atopic dermatitis, the lesions often spread to unaffected skin within 7-10 days. Over time, the vesicles progress into pustules and scabs, forming erosions that may result in scarring within 2-6 weeks [3].

Diagnosis relies on clinical presentation and HSV-DNA detection via PCR. The Tzanck test does not distinguish between HSV and varicella-zoster virus infections [4]. The differential diagnosis includes impetigo, primary varicella infection, disseminated herpes zoster, hand-foot-and-mouth disease, eczema coxsackium, disseminated molluscum contagiosum, cellulitis, and erysipelas.

Complications include herpetic keratoconjunctivitis and bacterial and viral superinfection, notably Staphylococcus and Molluscum contagiosum. Misdiagnosis, often as impetigo, can lead to severe complications and even death. In severe cases, viral dissemination can lead to meningitis and encephalitis, with mortality rates reaching up to 75% in the absence of targeted antiviral therapy. Due to frequent secondary infections, mainly by Staphylococcus, antibiotics are commonly included in the initial EH treatment [5]. Timely diagnosis and treatment with acyclovir are crucial to avert these outcomes.

## Conclusions

This case highlights the intricate interplay between AD and HSV infection, shedding light on the enigmatic nature of such presentations. All internists should be aware of this presentation. Timely diagnosis and appropriate management can prevent severe complications and decrease morbidity.
